# Supplementary material for: Prognostic Significance of Endocrine-Related Adverse Events in Patients with Melanoma, Non-Small Cell Lung Cancer and Urothelial Cancer After Treatment with Immune Checkpoint Inhibitors: A Systematic Review and Meta-Analysis
Source: Cancers (Basel). 2025 Nov 17;17(22):3675. doi: 10.3390/cancers17223675 (PMC12651665; doi:10.3390/cancers17223675)
Supplement: Supplementary file 1 [file cancers-17-03675-s001.zip › cancers-3958388-supplementary.pdf]

Supplementary Table S1. Detailed characteristics of and information retrieved from the included studies.

First author, Publication Year	Study Design	Country	Follow-up time (months)	Patients treated	Tumor type	ICIs types	Included irAEs types
Ahn, 2018	Retrospective	South Korea	17.0	155	NSCLC	PD-1	Hypothyroidism, Hypophysitis, Adrenal insufficiency
Basak, 2019	Retrospective	Netherlands	14.9	168	NSCLC	PD-1	Thyroiditis
Bellmunt, 2017	Prospective	USA	14.1	542	Urothelial	PD-1	Thyroiditis
Brahmer, 2015	Prospective	USA, Italy, Netherlands, Germany, Russia, Poland, Spain, Czechia	11.0	260	NSCLC	PD-1	Hypothyroidism
Campredon, 2019	Retrospective	France	9.0	105	NSCLC	PD-1	Thyroiditis
Chmielewska, 2021	Retrospective	Poland	36.0	35	NSCLC	PD-1	Thyroiditis, Hypophysitis, Adrenal insufficiency
Cortellini, 2018	Retrospective	Italy	12.7	559	NSCLC	PD-1	Thyroiditis
Cortellini, 2020	Retrospective	Italy, UK, Switzerland, Netherlands	17.2	877	NSCLC	PD-1	Thyroiditis
Cortijo- Cascajares, 2021	Retrospective/Prospective	Spain	63.0	75	NSCLC	PD-1	Hypothyroidism
Dick, 2016	Retrospective	Germany	12.4	86	Melanoma	CTLA-4	Hypophysitis
Eggermont, 2015	Prospective	France, USA, Italy, Denmark, Canada, Belgium	32.9	475	Melanoma	CTLA-4	Hypophysitis
Faje, 2018	Retrospective	USA	11.5	154	Melanoma	CTLA-4	Hypophysitis
Fradet, 2019	Retrospective	Canada	27.7	270	Urothelial	PD-1	Hypothyroidism
Fujisawa, 2017	Retrospective	Japan	7.3	60	Melanoma	PD-1, CTLA-4	Thyroiditis, Hypophysitis
Galsky, 2020	Prospective	USA, UK, Italy, Japan, Australia, Brasil, Greece, Japan, Taiwan, Turkey, South Korea, Austria, Germany, Slovenia	11.8	1213	Urothelial	PD-L1	Hypothyroidism
Grande, 2024	Retrospective	Spain, Germany, Austria, Greece, Portugal, Italy, USA, UK,	24.2	851	Urothelial	PD-L1	Thyroid disorders, Adrenal insufficiency, Pancreatitis

		Canada, Australia etc					
Grangeon, 2017	Retrospective	France	14.0	270	NSCLC	PD-L1	Thyroiditis
Haratani, 2018	Retrospective	Japan	11.1	134	NSCLC	PD-1	Hypophysitis, Hypothyroidism, Thyroiditis
Hayasi, 2024	Retrospective	Japan	11.5	1002	NSCLC	PD-L1	Thyroid disorders, Hypophysitis, Adrenal insufficiency, DM I
Herbst, 2015	Prospective	USA, Spain, Brazil, Chile, South Korea, Netherlands	19.0	345	NSCLC	PD-1	Thyroid disorders, Addrenal insufficiency, Hypophysitis, DM I
Hodi, 2018	Retrospective	USA, France, Germany, Italy, Poland, Switzerland	7.8	945	Melanoma	PD-1, CTLA-4	Thyroid disorders, Adrenal insufficiency, Hypophysitis, Pancreatitis
Hosoya, 2020	Prospective	Japan	1.5	76	NSCLC	PD-1	Hypothyroidism
Langer, 2016	Prospective	USA, Taiwan	14.0	60	NSCLC	PD-1	Thyroid disorders, Adrenal insufficiency
Lim, 2020	Retrospective	South Korea	30.1	299	NSCLC	PD-1	Hypothyroidism
Lisberg, 2018	Retrospective	USA	2.3	97	NSCLC	PD-1	Hypothyroidism
Muir, 2021	Retrospective	Australia	11.3	1246	Melanoma	PD-1, CTLA-4	Thyroiditis
Narita, 2021	Retrospective	Japan	17.0	121	Urothelial	PD-1	Thyroiditis, Adrenal insufficiency
Ohe, 2022	Retrospective	Japan	12.0	2570	NSCLC	PD-L1	DM I, Pancreatitis, Thyroiditis, Hypophysitis, Adrenal insufficiency
Osorio, 2016	Retrospective	USA	30.0	48	NSCLC	PD-1	Hypothyroidism, Hyperthyroidism
Paz-Ares, 2021	Prospective	Spain, USA, South Korea, Greece, France, Romania, Chile, Australia, Canada, Argentina etc	54.8	1739	NSCLC	PD-1, CTLA-4	Hypothyroidism
Peiro, 2018	Prospective	Spain	13.2	73	NSCLC	PD-1, CTLA-4	Thyroiditis
Riciuti, 2018	Retrospective	Italy	26.0	195	NSCLC	PD-1	Hypophysitis, Hypothyroidism, Hyperthyroidism, Adrenal insufficiency
Rubino, 2021	Retrospective	Italy	18.5	251	NSCLC	PD-1	Thyroiditis,

							Adrenal insufficiency, DM I
Rogado, 2019	Prospective	Spain	30.4	77	NSCLC	PD-1	Thyroid disorders, Hypophysitis, Adrenal insufficiency, DM I
Sakakida, 2019	Retrospective	Japan	4.3	150	Melanoma	PD-1	Thyroiditis
Schachter, 2017	Retrospective	Australia, Austria, Chile, Colombia, USA, France, UK, Germany, Israel, Netherlands etc	22.9	811	Melanoma	PD-1, CTLA-4	Hypothyroidism
Seejore, 2021	Retrospective	UK	16.7	189	NSCLC	PD-1, CTLA-4	Thyroiditis, Hypophysitis, Adrenal insufficiency, DM I
Serna-Higuita, 2021	Retrospective	Germany	24.0	319	Melanoma	PD-1, CTLA-4	Thyroiditis, Hypophysitis
Suo, 2020	Retrospective	Canada	24.0	186	Melanoma	PD-1	Hypothyroidism, Hypophysitis, Adrenal insufficiency, DM I
Wei, 2019	Retrospective	UK	7.0	51	Melanoma	PD-1, CTLA-4	Hypophysitis, Hypothyroidism
Wu, 2018	Retrospective	Taiwan	9.1	49	Melanoma	PD-1	Thyroiditis, Hypophysitis, Adrenal insufficiency
Yamauchi, 2019	Retrospective	Japan	15.0	200	NSCLC	PD-1	Thyroiditis
Zhang, 2022	Retrospective	China	6.5	11	NSCLC	PD-1	Thyroiditis

Supplementary Table S2. Risk of Bias (per study)

Author	Year	Country/ Region	Cancer	Study design	ICI class	Randomiza-tion/Confound-ing	Deviations from intended interventions	Measurement of outcomes	Selection of re-ported results	Overall RoB
Ahn	2018	South Korea	NSCLC	Retrospective cohort	PD-1	Moderate	Low	Low	Low	Moderate
Basak	2019	Netherlands	NSCLC	Retrospective cohort	PD-1	Moderate	Low	Low	Low	Moderate
Bellmunt	2017	USA	Urothelial	Randomized trial	PD-1	Low	Low	Low	Low	Low
Brahmer	2015	Multi (EU/US)	NSCLC	Prospective trial (non-rand.)	PD-1	Moderate	Low	Low	Low	Low
Campredon	2019	France	NSCLC	Retrospective cohort	PD-1	Moderate	Low	Low	Low	Moderate
Chmielewska	2021	Poland	NSCLC	Retrospective cohort	PD-1	High	Moderate	Moderate	Moderate	High
Cortellini	2018	Italy	NSCLC	Retrospective multicenter	PD-1	Moderate	Low	Low	Low	Moderate
Cortellini	2020	It-aly/UK/CH/NL	NSCLC	Retrospective multicenter	PD-1	Moderate	Low	Low	Low	Moderate
Cortijo-Cas-cajares	2021	Spain	NSCLC	Retro/ Prospective	PD-1	Moderate	Low	Moderate	Low	Moderate
Dick	2016	Germany	Melanoma	Retrospective cohort	CTLA-4	Moderate	Low	Low	Low	Moderate
Eggermont	2015	Multi (EU/NA)	Melanoma	Randomized trial	CTLA-4	Low	Low	Low	Low	Low
Faje	2018	USA	Melanoma	Retrospective cohort	CTLA-4	Moderate	Low	Low	Low	Moderate
Fradet	2019	Canada	Urothelial	Retrospective cohort	PD-1	Low	Low	Low	Low	Low
Fujisawa	2017	Japan	Melanoma	Retrospective cohort	PD-1/CTLA-4	Moderate	Low	Moderate	Low	Moderate

Author	Year	Country/ Region	Cancer	Study design	ICI class	Randomiza-tion/Confound-ing	Deviations from intended interventions	Measurement of outcomes	Selection of re-ported results	Overall RoB
Galsky	2020	Multi (global)	Urothelial	Randomized trial	PD-L1	Low	Low	Low	Low	Low
Grande	2024	Multi (EU/NA)	Urothelial	Retrospective multicenter	PD-L1	Low	Low	Low	Low	Low
Grangeon	2017	France	NSCLC	Retrospective cohort	PD-L1	Low	Low	Low	Low	Low
Haratani	2018	Japan	NSCLC	Retrospective cohort	PD-1	Low	Low	Low	Low	Low
Hayasi	2024	Japan	NSCLC	Retrospective cohort	PD-L1	Low	Low	Low	Low	Low
Herbst	2015	Multi (global)	NSCLC	Prospective trial (non-rand.)	PD-1	Low	Low	Low	Low	Low
Hodi	2018	Multi (EU/US)	Melanoma	Retrospective cohort	PD-1/CTLA-4	Low	Low	Low	Low	Low
Hosoya	2020	Japan	NSCLC	Prospective (non-rand.)	PD-1	Moderate	Low	Low	Low	Moderate
Langer	2016	USA/Taiwan	NSCLC	Prospective (non-rand.)	PD-1	Low	Low	Low	Low	Low
Lim	2020	South Korea	NSCLC	Retrospective cohort	PD-1	Low	Low	Low	Low	Low
Lisberg	2018	USA	NSCLC	Retrospective cohort	PD-1	High	Moderate	Moderate	Moderate	High
Muir	2021	Australia	Melanoma	Retrospective cohort	PD-1/CTLA-4	Low	Low	Low	Low	Low
Narita	2021	Japan	Urothelial	Retrospective cohort	PD-1	Low	Low	Low	Low	Low
Ohe	2022	Japan	NSCLC	Retrospective cohort	PD-L1	Low	Low	Low	Low	Low
Osorio	2016	USA	NSCLC	Retrospective cohort	PD-1	High	Moderate	Moderate	Moderate	High

Author	Year	Country/ Region	Cancer	Study design	ICI class	Randomiza-tion/Confound-ing	Deviations from intended interventions	Measurement of outcomes	Selection of re-ported results	Overall RoB
Paz-Ares	2021	Multi (global)	NSCLC	Randomized trial	PD-1/CTLA-4	Low	Low	Low	Low	Low
Peiro	2018	Spain	NSCLC	Prospective (non-rand.)	PD-1/CTLA-4	Moderate	Low	Low	Low	Moderate
Riciuti	2018	Italy	NSCLC	Retrospective cohort	PD-1	Low	Low	Low	Low	Low
Rubino	2021	Italy	NSCLC	Retrospective cohort	PD-1	Low	Low	Low	Low	Low
Rogado	2019	Spain	NSCLC	Prospective (non-rand.)	PD-1	Low	Low	Low	Low	Low
Sakakida	2019	Japan	Melanoma	Retrospective cohort	PD-1	Low	Low	Low	Low	Low
Schachter	2017	Multi (global)	Melanoma	Retrospective cohort	PD-1/CTLA-4	Low	Low	Low	Low	Low
Seejore	2021	UK	NSCLC	Retrospective cohort	PD-1/CTLA-4	Low	Low	Low	Low	Low
Serna-Higuita	2021	Germany	Melanoma	Retrospective cohort	PD-1/CTLA-4	Moderate	Low	Low	Low	Moderate
Suo	2020	Canada	Melanoma	Retrospective cohort	PD-1	Moderate	Low	Low	Low	Moderate
Wei	2019	UK	Melanoma	Retrospective cohort	PD-1/CTLA-4	Moderate	Low	Low	Low	Moderate
Wu	2018	Taiwan	Melanoma	Retrospective cohort	PD-1	High	Moderate	Moderate	Moderate	High
Yamauchi	2019	Japan	NSCLC	Retrospective cohort	PD-1	Low	Low	Low	Low	Low
Zhang	2022	China	NSCLC	Retrospective cohort	PD-1	Moderate	Low	Low	Low	Moderate
Zhang	2025	China	NSCLC	Multicenter retrospective	PD-1	Moderate	Low	Low	Low	Moderate

Supplementary Table S3. Certainty of Evidence (GRADE Summary of Findings)

Outcome	No. of studies	Designs included	Risk of bias	Inconsistency	Indirectness	Imprecision	Publication bias	Overall GRADE	Key comments
**Overall Survival (OS)**	43	RCTs + observational	☐	☑ (moderate heterogeneity)	☐	☐	☑ (minor funnel-plot asymmetry)	**Moderate**	Consistent direction and magnitude of effect (HR ≈ 0.60); sensitivity analyses robust; small risk of publication bias.
**Progression-Free Survival (PFS)**	43	RCTs + observational	☐	☐–☑ (mild heterogeneity)	☐	☐	☐	**Moderate to High**	Stable and reproducible association across tumor types (HR ≈ 0.61); results coherent between study designs.
**Endocrine irAEs (any grade)**	43	Mixed	☑	☑	☐	☑	☐	**Low to Moderate**	Considerable variability in definitions and ascertainment; wide confidence intervals; moderate between-study heterogeneity.
**Grade 3–4 endocrine irAEs**	24	Mixed	☑	☑	☐	☑	☐	**Moderate**	Higher incidence with anti-PD-L1 agents in urothelial cancer and with pembrolizumab in NSCLC/melanoma; precision limited by subgroup size.
**Thyroid dysfunction (all)**	38	Mixed	☐	☐	☐	☐	☐	**High**	Very consistent findings across cancer types and ICI classes; minimal heterogeneity and narrow CIs.
**Hypophysitis**	19	Mixed	☑	☑	☐	☑	☐	**Moderate**	Small sample sizes and variable diagnostic criteria; substantial heterogeneity in onset timing and assessment.
**Adrenal insufficiency**	15	Mixed	☑	☑	☑	☑	☐	**Low**	Rare outcome; imprecise estimates; inconsistent case definition and reporting across studies.
**Autoimmune diabetes (DM1)**	10	Mixed	☑	☑	☐	☑	☐	**Low**	Very infrequent event; wide CIs and reporting variability limit certainty; effect direction remains consistent but imprecise.

Appendix B

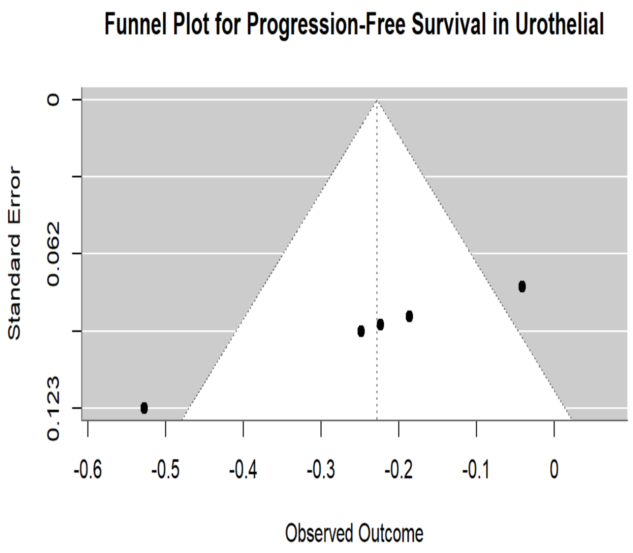
Funnel Plot for Progression-Free Survival in Urothelial
Standard Error
0
0.062
0.123
-0.6
-0.5
-0.4
-0.3
-0.2
-0.1
0
Observed Outcome

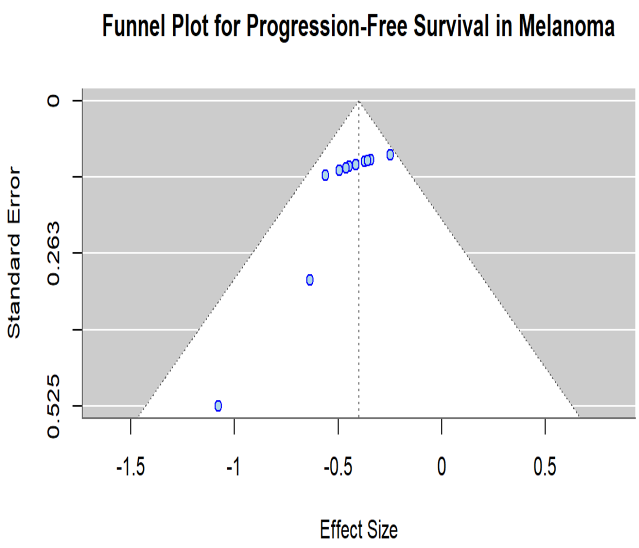
Funnel Plot for Progression-Free Survival in Melanoma
Standard Error
0
0.263
0.525
-1.5
-1
-0.5
0
0.5
Effect Size

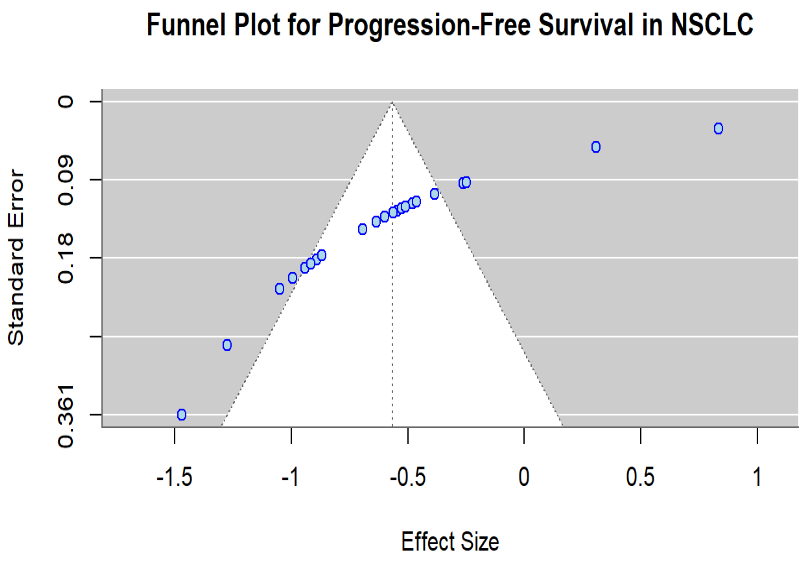
Funnel Plot for Progression-Free Survival in NSCLC
Standard Error
0
0.09
0.18
0.361
-1.5
-1
-0.5
0
0.5
1
Effect Size

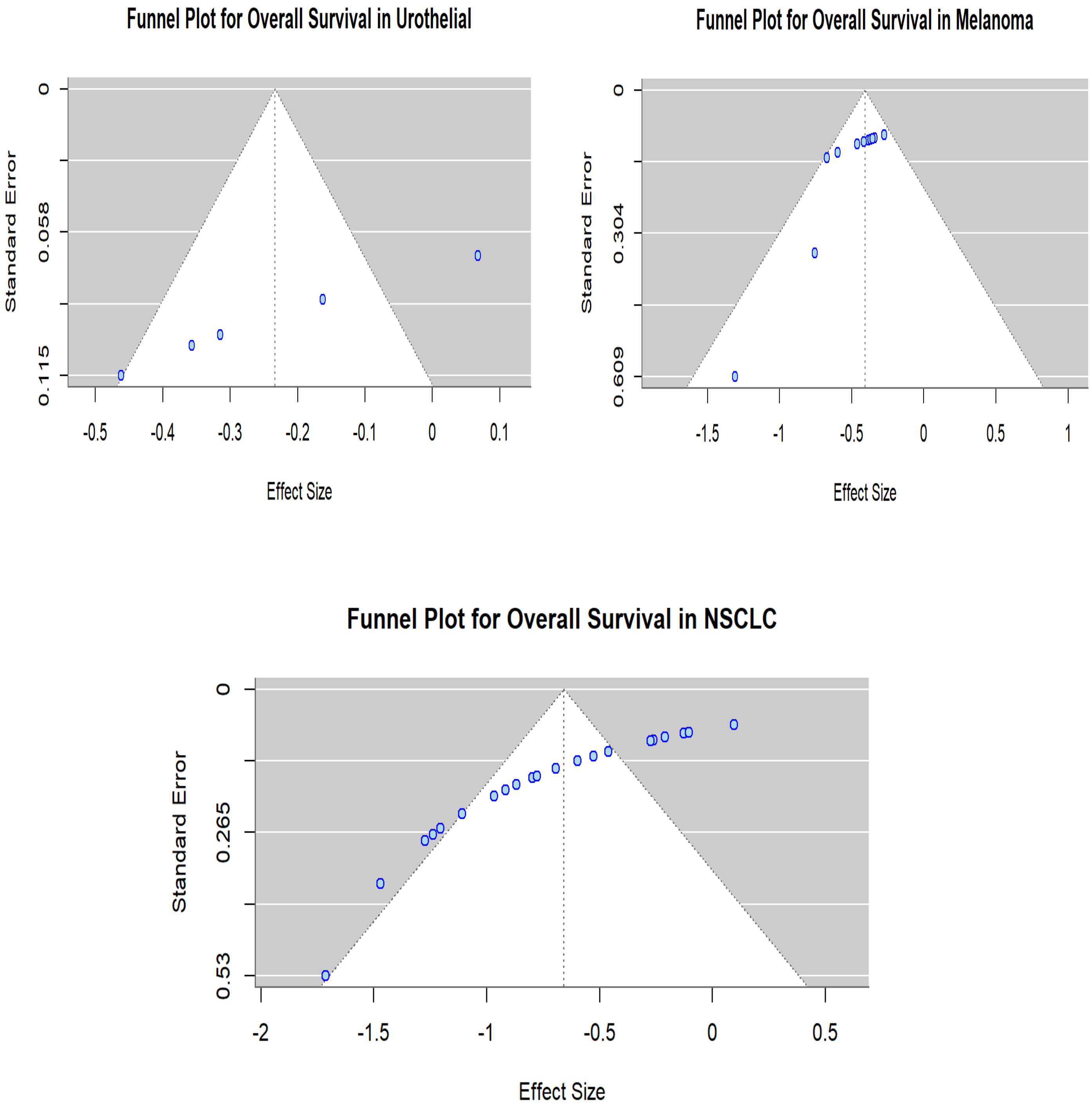

Supplementary Figure S1. Funnel plots assessing publication bias for studies included in the analysis for patients with melanoma, NSCLC and urothelial cancer.

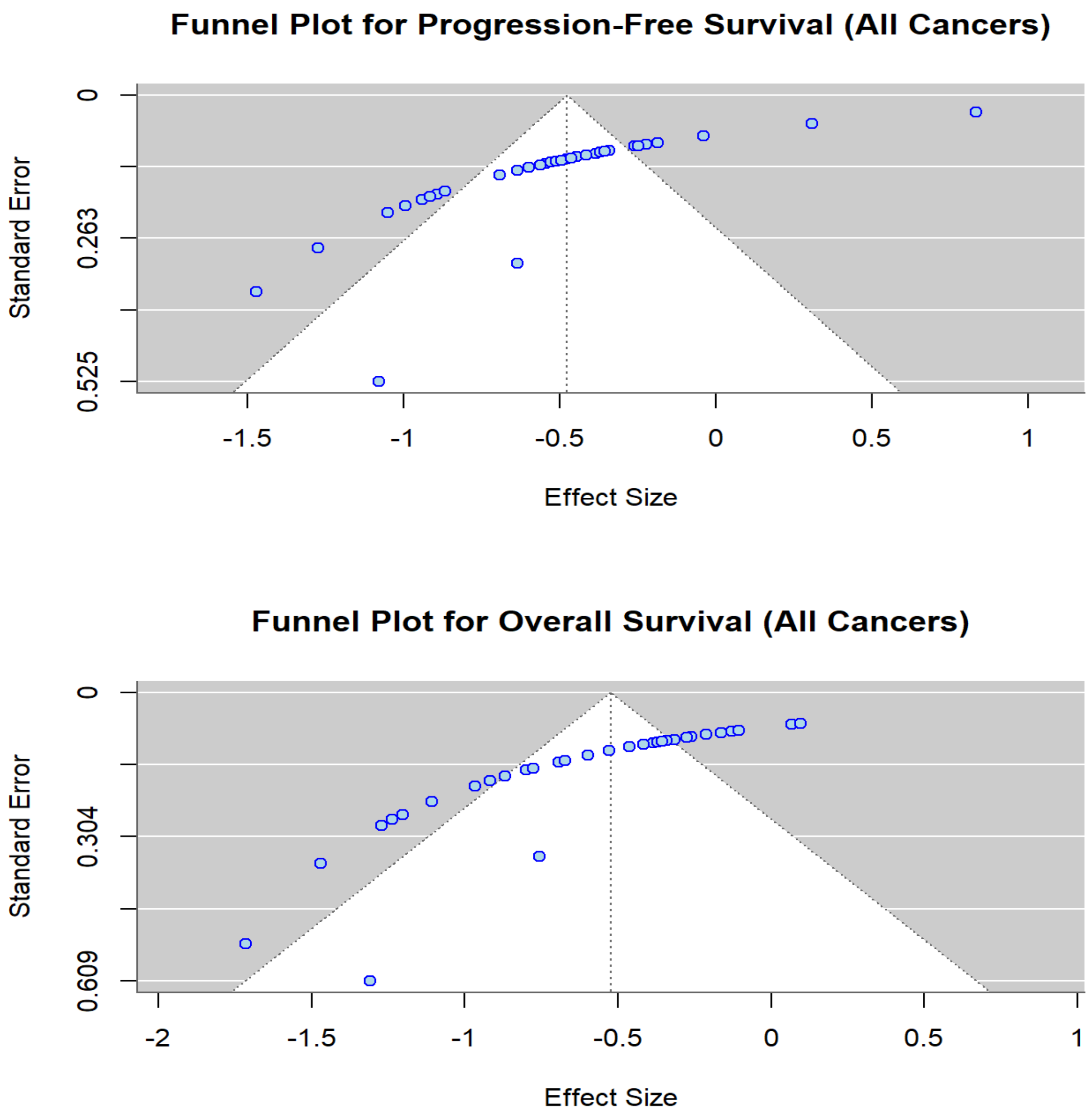

Supplementary Figure S2. Funnel plots assessing overall publication bias for studies included in the analysis for patients with melanoma, NSCLC and urothelial cancer.

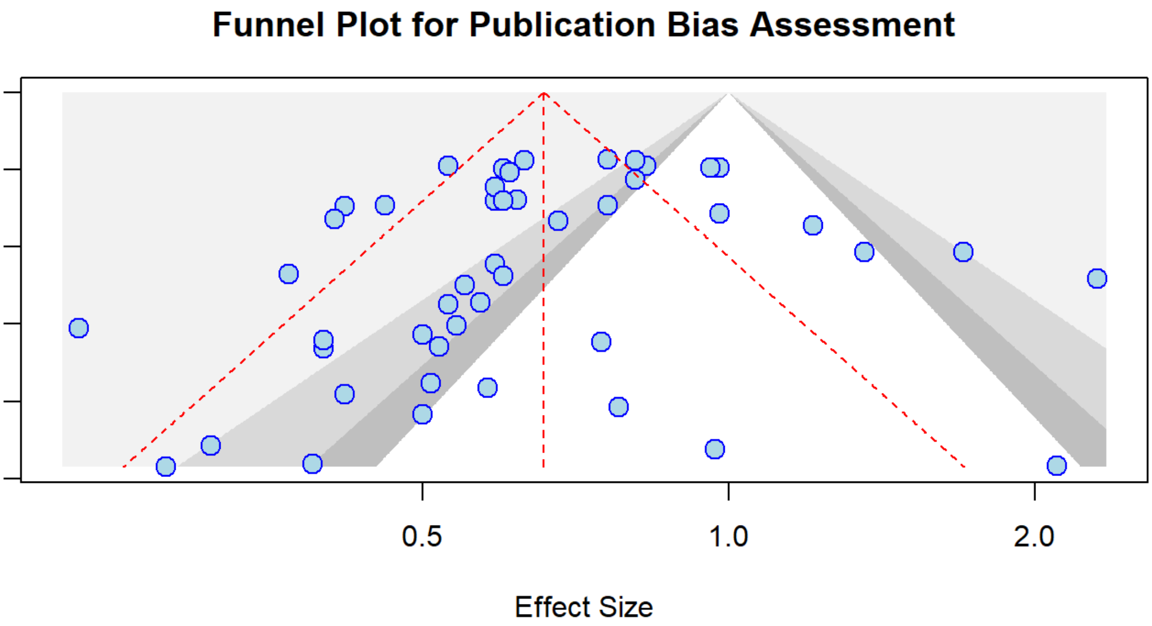

Supplementary Figure S3. Contour funnel plot assessing publication bias for all studies included for melanoma, NSCLC and urothelial cancer.

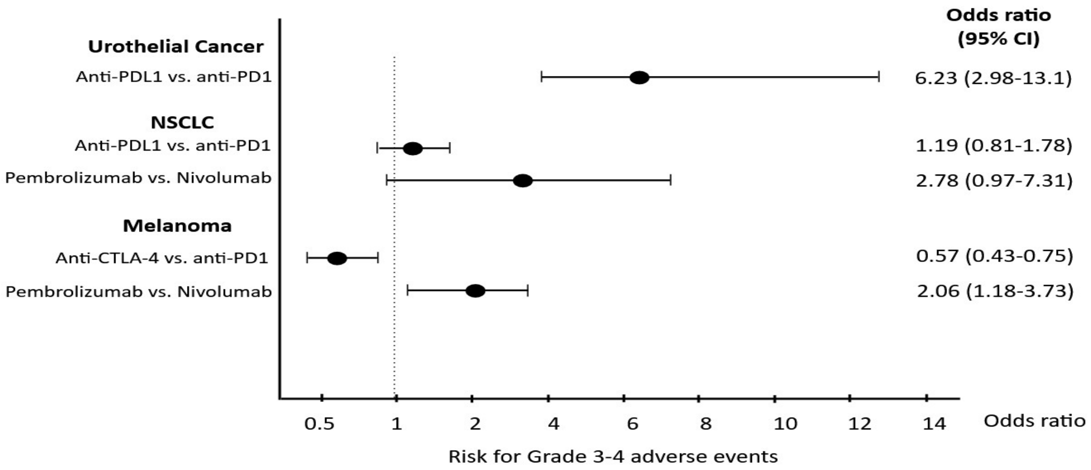

Supplementary Figure S4. Pooled odds ratios of risk for grade 3-4 endocrine adverse events in patients with urothelial cancer, NSCLC and melanoma. CI, confidence interval.

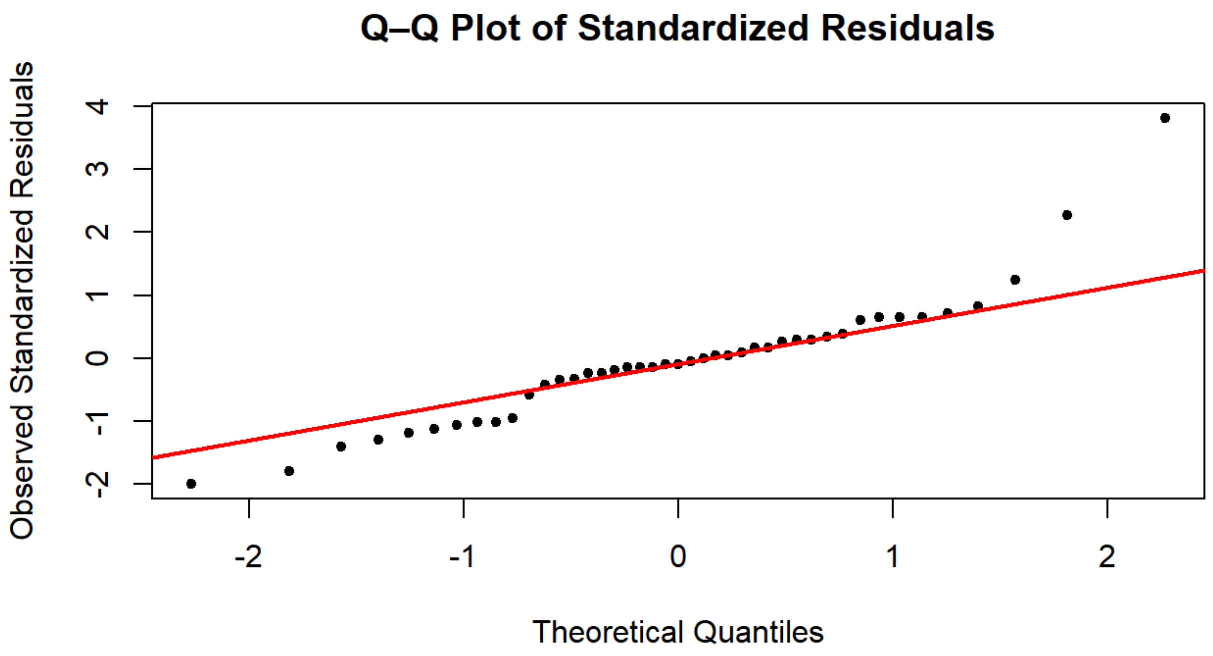

Supplementary Figure S5. Q-Q Plot of Standarised Residuals. The plot displays the distribution of residuals from the univariate meta-regression model examining the relationship between log-transformed hazard ratios (logHR) for progression-free survival (PFS) and endocrine adverse event odds. The vertical line represents the mean residual.
